# Effects of dietary protease supplementation on *in vitro* soybean meal protein, dry matter digestibility, and productive performance in starter-to-finisher pigs

**DOI:** 10.14202/vetworld.2024.2185-2192

**Published:** 2024-09-28

**Authors:** Phubet Satsook, Surapan Jitviriyanon, Anchalee Khongpradit, Sirinapa Chungopast, Chanwit Kaewtapee, Nitipong Homwong

**Affiliations:** 1 Department of Animal Science, Faculty of Agriculture at Kamphaeng Saen, Kasetsart University Kamphaeng Saen Campus, Nakhon Pathom, 73140 Thailand; 2 National Swine Research and Training Center, Faculty of Agriculture at Kamphaeng Saen, Kasetsart University, Kamphaeng Saen Campus, Nakhon Pathom, 73140, Thailand; 3 Department of Soil Science, Faculty of Agriculture at Kamphaeng Saen, Kasetsart University Kamphaeng Saen Campus, Nakhon Pathom, 73140 Thailand; 4 Department of Animal Science, Faculty of Agriculture, Kasetsart University, 50 Ngam Wong Wan Rd., Latyao, Chatuchak, Bangkok, 10900 Thailand

**Keywords:** digestibility, performance, pig, production, protease

## Abstract

**Background and Aim:**

Pig industries are currently facing a crisis in terms of protein and energy costs. Proteases were used to increase protein digestibility and metabolizable energy (ME) in diets. This study evaluated the effects of protease supplementation on *in vitro* protein digestibility and productive performance in starter-to-finisher pigs.

**Materials and Methods:**

A total of 691 starter pigs were randomly allocated into three dietary treatments using a randomized complete block design. Diets were provided in three phases according to body weight (BW): Starter, grower, and finisher phases. Each phase was fed for 30, 60, and 24 days of treatment diets as T1: basal diet and T2 and T3: the basal diet supplemented with 240 ppm protease reduced by 50 kcal/kg ME plus 1% crude protein (CP) and by 100 kcal/kg ME plus 2% CP, respectively. Protease and *in vitro* protein digestibility were measured. BW and feed intake were recorded to calculate the average daily gain (ADG), average daily feed intake (ADFI), feed-to-gain (F:G), and gain-to-feed (G:F) ratios.

**Results:**

There were no significant differences (p > 0.05) in the percentage of *in vitro* protein digestibility between the groups with and without protease supplementation. In the finisher phase, T2 had lower (p < 0.05) ADFI and F:G than T1 and T3. Overall, T3 had lower (p < 0.05) ADG, ADFI, and F:G than T1 and T2.

**Conclusion:**

Protease supplementation significantly affects protein digestibility. Supplementing basal diets with 240 ppm protease reduced ME by 50 kcal/kg and CP by 1% without affecting ADG, ADFI, F:G, and G:F ratios for starter-to-finisher pigs.

## Introduction

Protein and energy nutrients are the major cost components in producing feed [1]. Recently, protein and energy feed costs have consistently increased [2]. Whenever lowering feed cost, this may influence feed quality or growth efficiency in pigs. As the pig production industry evolves with high feed ingredient costs and varied feed ingredient quality, focusing on increasing nutrient digestibility, reducing feed costs, and increasing environmental sustainability is becoming increasingly important [1]. Feed enzyme supplementation has been suggested as a nutritional and environmental strategy for enhancing nutrient digestibility, improving gain efficiency, reducing cost, improving consistency of feed ingredients, and helping to maintain gut health and a better environment [3]. Exogenous proteases have been employed in the form of several enzyme admixtures for almost two decades, thus becoming an attractive strategy available in the pig production industry [4, 5].

Proteases, protein-degrading enzymes, have been suggested to be implemented in non-ruminant diets, such as those in pigs. Dietary protease highly degrades cross-linked and recalcitrant structural proteins, thereby improving the digestibility and nutritional value of dietary proteins [6]. Exogenous dietary proteases comprise a range of endo-proteases, including pepsin (EC.3.4.21.4), trypsin (EC 3.4.21.4), chymotrypsin (EC 3.4.21.1), and elastase (EC 3.4.21.36) [1]. Pepsin is an aspartic endo-protease with broad specificity and is an acid protease suitable for the gastric phase of animals. Trypsin, chymotrypsin, and elastase are all endo-proteases of the serine protease family and work optimally at pH around 7–9, suitable for the neutral environment in the small intestine (duodenum, jejunum, and ileum) [1]. A previous study by Park *et al*. [7] found that 1,125,000 protease unit/kg feed included in diets based on corn and soybean meal increased the apparent total tract digestibility and ileal digestibility of dry matter, crude protein (CP), and metabolizable energy (ME) in weaned pigs. These effects were confirmed when grower-to-finisher pigs showed significant improvements in final body weight (BW), average daily gain (ADG), and the gain-to-feed ratio (G:F) [8]. Supplementation with 300,000 protease unit/kg feed provided better villus height, improved the ratio between villus height and crypt depth, and increased the number of goblet cells in both newly weaned and starter pigs [9, 10]. In addition, an inclusion rate of 250 ppm of protease in the form of coated compound enhanced nitrogen utilization and decreased manure nitrogen output [11]. An advantage was also observed when protease was supplemented in a low-protein diet (1%–2% CP reduced from basal diet), resulting in better CP digestibility and productive performance in weaning-to-finishing pigs [12]. These studies suggested that the potential of protease supplementation in diets consequently improved productive performance in pigs fed either a normal or low-protein diet. Exogenous protease was implemented to reduce protein levels, primarily in monogastric animals [8, 12, 13]. On the other hand, energy reduction has been studied mostly in broilers [14, 15], while a trial of energy reduction was rarely conducted in pigs.

Therefore, this study aimed to determine the effects of protease on *in vitro* soybean meal protein and dry matter digestibility and on the productive performance of starter-to-finisher pigs while simultaneously lowering ME and CP levels in soybean meal-broken rice basal diets.

## Materials and Methods

### Ethical approval

This study was approved by the Institutional Animal Care and Use Committee of Kasetsart University, Thailand (ACKU64-AGK-010).

### Study period and location

The study was conducted from June-2021 to November-2021 at a commercial farm located in western Thailand.

### Study design

We used the R program to determine the sample size. The number of all 691 pigs were not experimental units. However, we considered pens as the experimental units. There were three treatments from all 18 pens, six pens for each treatment. We used k = 3, n = 6, f = 0.82, and significance level = 0.05, where k is the number of treatments, n is the number of experimental units for each treatment, and f is an effect size. Therefore we obtained the power of the test being 0.8088, which was satisfied for our assumption.

We used 691 starter pigs (50% Duroc × (25% Landrace × 25% Yorkshire]) with an average BW of 22–28 kg in this study. Of the 18 pens in a single house, 15 each contained 39 pigs, two each contained 38 pigs, and one contained 30 pigs. To reduce stress, stock density was limited to 39 pigs per pen (dimension = 5 × 8 m^2^, equivalent to 1 pig m^-2^). Mixed-sex piglets were allocated blocking by recruitment week using a randomized complete block design and were randomly assigned to three treatment groups (T1, T2, and T3). Initial BWs of T1, T2, and T3 were 23.36 ± 0.62, 27.90 ± 0.57, and 20.45 ± 0.57 kg, respectively. Pigs were housed in an evaporative cooling system.

The protease DigeGrain Pro 6^®^ (Advanced Enzyme Technologies Ltd., West Thane, Maharashtra, India) with 25,000,000 units/kg product was 240 ppm, equivalent to 6,000 units/kg feed. The protease DigeGrain Pro 6^®^, an endo-protease produced by a selected strain of *Bacillus licheniformis*, was used in this study with support from Union Castap Co., Ltd. (Bangkok, Thailand).

The experimental diets were provided into three phases according to BW as starter, grower, and finisher diets. As a starter diet (22–60 kg), T1 was formulated as 3,300 kcal/kg ME with 19% CP, T2 as 3,250 kcal/kg ME with 18% CP plus 240 ppm protease, and T3 as 3,200 kcal/kg ME with 17% CP plus 240 ppm protease. For the grower diet (61–100 kg), T1 was formulated as 3,150 kcal/kg ME with 18% CP, T2 as 3,100 kcal/kg ME with 17% CP plus 240 ppm protease, and T3 as 3,050 kcal/kg ME with 16% CP plus 240 ppm protease. As a finisher diet (101–120 kg), T1 was formulated as 3,000 kcal/kg ME with 17% CP, T2 as 2,950 kcal/kg ME with 16% CP plus 240 ppm protease, and T3 as 2,900 kcal/kg ME with 15% CP plus 240 ppm protease. The experimental diets were developed using FeedLIVE^®^ Version 1.61 (Live Informatics Co., Ltd. Nonthaburi, Thailand) and met or exceeded the National Research Council [16]. The ingredient composition and calculated nutritional values for each period are shown in Table-1 and were supplied *ad libitum* for approximately 19 weeks. Pigs had free access to water throughout the study.

**Table-1:** Ingredient compositions and calculated nutrients of the experimental diets (as-fed basis).

Ingredient	Starter			Grower			Finisher		
		
	T1	T2	T3	T1	T2	T3	T1	T2	T3
Broken rice	30.00	30.00	30.00	15.00	15.00	15.00	10.00	10.00	10.00
Tapioca meal (70%)	18.64	22.09	25.44	20.00	20.00	20.00	25.00	25.06	31.76
Rice bran	15.00	15.00	15.00	23.96	21.51	18.41	24.72	23.87	10.00
Extracted rice bran	-	-	-	10.00	16.11	22.95	15.00	19.62	29.11
Soybean oil	3.03	2.05	1.09	3.06	3.00	3.00	1.27	1.00	1.00
SBM (45.5%)	27.98	25.34	22.83	23.98	20.00	15.93	20.80	17.00	15.00
Fish meal (56%)	1.50	1.50	1.50	-	-	-	-	-	-
L-Lysine	0.24	0.33	0.38	0.23	0.31	0.38	0.12	0.18	0.18
DL-Methionine	0.18	0.21	0.22	0.20	0.21	0.22	0.13	0.11	0.10
L-Threonine	0.10	0.14	0.16	0.13	0.16	0.19	0.09	0.10	0.05
Choline chloride (60%)	0.01	0.01	0.01	-	-	-	-	-	-
Monodicalcium-phosphate	1.15	1.19	1.25	1.32	1.66	2.10	1.21	1.33	0.79
Calcium carbonate (CaCO_3_)	0.88	0.86	0.83	0.94	0.86	0.65	1.00	1.06	1.25
Salt (NaCl)	0.37	0.37	0.37	0.40	0.40	0.40	0.40	0.40	0.50
Zinc Oxide	0.25	0.25	0.25	-	-	-	-	-	-
^1^PX Starter	0.25	0.25	0.25	-	-	-	-	-	-
^2^PX Grower	-	-	-	0.25	0.25	0.25	-	-	-
^3^PX Finisher	-	-	-	-	-	-	0.25	0.25	0.25
^4^DigeGrainPro	-	0.024	0.024	-	0.024	0.024	-	0.024	0.024
^5^OptiPhose	0.01	0.01	0.01	0.01	0.01	0.01	0.01	0.01	0.01
^6^HostazymX	0.01	0.01	0.01	0.01	0.01	0.01	0.01	0.01	0.01
^7^Dx	0.40	0.40	0.40	0.50	0.50	0.50	-	-	-
Total	100.00	100.00	100.00	100.00	100.00	100.00	100.00	100.00	100.00
Nutrient, %									
ME, kcal/kg	3,300.00	3,250.00	3,200.00	3,150.00	3,100.00	3,050.00	3,000.00	2,950.00	2,900.00
Crude protein	19.00	18.00	17.00	18.00	17.00	16.00	17.00	16.00	15.00
Crude fat	5.85	4.86	3.89	6.99	6.59	6.17	5.31	4.93	3.00
Crude fiber	4.14	4.06	3.97	5.91	6.12	6.35	6.48	6.68	6.48
Total calcium	0.90	0.90	0.90	0.85	0.89	0.87	0.87	0.90	0.90
Total phosphorus	0.76	0.75	0.75	0.98	1.09	1.21	1.02	1.07	0.89
Available phosphorus for swine	0.45	0.45	0.46	0.48	0.54	0.61	0.47	0.49	0.40
Sodium	0.22	0.22	0.22	0.22	0.22	0.22	0.22	0.22	0.23
Total lysine	1.20	1.20	1.18	1.10	1.15	1.13	0.95	0.93	0.88
Total methionine	0.47	0.49	0.48	0.47	0.48	0.48	0.38	0.36	0.34
Total methionine and cysteine	0.74	0.74	0.72	0.72	0.73	0.71	0.62	0.58	0.56
Total threonine	0.78	0.78	0.76	0.75	0.77	0.75	0.67	0.63	0.56
Total tryptophan	0.24	0.22	0.21	0.21	0.21	0.19	0.19	0.17	0.16
Choline, mg/kg	1,614.64	1,543.43	1,475.63	1,323.88	1,263.88	1,202.71	1,242.77	1,189.36	1,078.39

^1^Dietary premix for starter per kilogram of feed content: vitamin A 15,000 IU, vitamin D3 3,000 IU, vitamin E 36 IU, vitamin K3 2.00 mg, vitamin B1 2.00 mg, vitamin B2 7.00 mg, vitamin B6 4.00 mg, vitamin B12 0.03 mg, pantothenic acid 16.00 mg, niacin 30.00 mg, folic acid 0.90 mg, biotin 0.15 mg, choline 0.30 g, ferrous 150.00 mg, copper 140.00 mg, manganese 50.00 mg, cobalt 0.25 mg, zinc 100.00 mg, iodine 2.00 mg, and selenium 0.25 mg. ^2^Dietary premix for growers per kilogram of feed content: vitamin A 10,000 IU, vitamin D3 3000.00 IU, vitamin E 60 IU, vitamin K3 2.50 mg, vitamin B1 1.50 mg, vitamin B2 5.00 mg, vitamin B6 3.0 mg, vitamin B12 0.025 mg, niacin 25.00 mg, folic acid 0.60 mg, biotin 0.15 mg, pantothenic acid 15.00 mg, ferrous 160.00 mg, copper 150.00 mg, manganese 60.00 mg, cobalt 0.60 mg, zinc 125.00 mg, iodine 1.20 mg, and selenium 0.25 mg. ^3^Dietary premix for finisher per kilogram of feed content: vitamin A 12,800 IU, vitamin D3 2,560 IU, vitamin E 24 IU, vitamin K3 1.13 mg, vitamin B1 1.60 mg, vitamin B2 3.20 mg, vitamin B6 2.08 mg, vitamin B12 0.02 mg, niacin 23.84 mg, folic acid 0.48 mg, biotin 0.10 mg, pantothenic acid 8.00 mg, ferrous 160.00 mg, copper 160.00 mg, manganese 50.00 mg, cobalt 1.00 mg, zinc 100.00 mg, iodine 1.00 mg, and selenium 0.30 mg. ^4^Protease as a treatment for experimental diet. ^5^Phytase enzymes in diets. ^6^Xylanase enzyme in experimental diets. ^7^Feed medication

All performance data records, and inventory data were digitized in PigLIVE^®^ Version 4.0 (Live Informatics Co., Ltd., Nonthaburi, Thailand) and Microsoft Excel^®^ 365 (Microsoft Corporation, Washington, USA). Individual pigs, amount of feed intake, and feed refusal in each pen were weighed and recorded four times as follows: (1) Received the pigs for experiment used as initial BW of starter phase; (2) the final BW of starter phase used as initial BW of grower phase; (3) the final BW of grower phase used as initial BW of finisher phase; and (4) the final BW of finisher phase used as the end of the study.

### Enzyme activity and protein levels

Pancreatin (50 mg pancreatin, porcine, grade IV, Sigma No. P-1790, USA) and Protease DigeGrain Pro 6^®^ (Advanced Enzyme Technologies Ltd.) were assayed for enzyme activity. Both powdered enzymes were diluted 1:10-fold in 0.1 M sodium phosphate buffer (pH = 7.5) solution. The 250 μL diluted enzymes were pipetted into 2% skim milk solution as a substrate and incubated at 37°C for 60 min to catalyze the enzyme reaction. The sample reaction between the enzymes and the substrate was stopped using 500 μL 10% trichloroacetic acid, and then, samples were immersed in ice for 1 h. After that, samples were centrifuged at 10,000× *g* for 10 min, and 0.5 mL of supernatant was mixed with 2.5 mL of solution C (copper sulfate 0.5 g and sodium citrate 1 g in distilled water 100 mL mixed with sodium carbonate 20 g and NaOH 4 g in distilled water 1 L, ratio 1:50 v/v), followed by 0.25 mL of solution D (Folin’s reagent: water; 1:1, v/v). The tested samples were incubated for 20 min, and the absorbance was measured at 750 nm.

A series of tyrosine standard solutions at different concentrations (20–100 μg/mL) were prepared. The interpretation was that one unit of the enzyme in the tested samples was equal to the amount of protease enzyme capable of releasing 1 μmol of tyrosine per milliliter [17, 18]. The total protein content of the enzyme was determined by the Bradford method [19] using the Bio-Rad Bradford reagent (USA). A series of dilutions of 2% bovine serum albumin standard solutions were prepared at a concentration of 0.125–2.0 mg/mL for the standard curve. Bradford assay was performed by adding 900 μL of Bradford reagent mixed with 30 μL of each standard solution or enzyme. The standard samples were incubated for 5 min in the dark, and the absorbance was measured at 595 nm. The specific activity of each enzyme was determined in units/g of protein (U/g).

### *In vitro* protein digestibility

Protein digestibility was calculated according to a previous report by Boisen and Ferna [20]. Briefly, 1 g ± 0.1 mg of soybean meal was weighted in 100 mL conical flasks. Twenty-five milliliters of phosphate buffer (0.1 M, pH 6.0) were added to a flask and mixed with magnetic stirring. Ten milliliters of HCl (0.2 M) were added to the mixture. pH 2 was adjusted using a 1 M HCl or 1 M NaOH solution. A freshly prepared pepsin solution (10 mg pepsin, porcine, 2,000 FIP/U/g, MERK No. 7190) was added to the mixture, and 0.5 g of chloramphenicol (Sigma No. C-0378) was employed to prevent bacterial growth. The flask was closed using a rubber stopper and incubated in a shaking water bath at 39°C for 6 h.

Ten milliliters of phosphate buffer (0.2 M, pH 6.8) and 5 ml of NaOH solution (0.6 M) were then added to the mixture. pH 6.8 was adjusted using a 1-M HCL or 1-M NaOH solution. One milliliter of freshly prepared pancreatin solution (50 mg pancreatin, porcine, Grade IV, Sigma No. P-1790) was added to the mixture. The flasks were closed with a rubber stopper and incubated in a shaking water bath at 39°C for 18 h.

Five milliliters of 20% sulphosalicylic acid were added to each sample. Solubilized proteins were precipitated after incubating at 25°C for 30 min. The undigested residues were then collected in a glass filter crucible filtration unit. All materials were transferred to a 1% sulphosalicylic acid. The undigested residues were dried at 80°C overnight after 2 consecutive washing with 10 mL of ethanol and acetone. *In vitro* digestibility was calculated as the difference between dry matter in the sample and undigested residues.

### Statistical analysis

Pig-day was defined as the number of days in which each pig remained in the pen at the end of the study (the final BW for finisher phase). Day-in-herd (DIH) was calculated by summing the pig days in each phase. Each DIH of the pigs was considered only within a pen; thereby, it defined as the pen DIH. The ADG determining the weight gain of individual live pigs was computed by subtracting the final pen total BW from the initial pen total BW and then dividing by the pen DIH. The average daily feed intake (ADFI) was determined by the total feed intake of the pen divided by the pen DIH. The BW gain was defined as the increase in the total BWs of either live or dead pigs within each pen from the initial BW. The G:F ratio, which was measured as gain efficiency, was calculated as the pen total BW gain divided by the pen total feed intake. In addition, G:F was calculated using ADG divided by ADFI. The feed-to-gain ratio (F:G), known as the feed conversion ratio, measured as feed efficiency, was the reciprocal of G:F.

The data were analyzed using a general linear model. Individual pigs were considered experimental animals for BW, ADG, ADFI, and DIH. However, the pens were considered the experimental unit for F:G and G:F. Since the initial BW was not homogenous, the final BW was adjusted using the residual of the initial BW (the residual adjusted treatment) through analysis of covariance (ANCOVA). The statistical model of the covariates was as follows:

Y_ijk_ = μ + CO + β_i_ + T_j_ + e_ijk_,

where Y_ijk_ is the dependent variable in diet *j* for block *i*; μ is the overall mean; CO is the covariate, the initial BW used to adjust treatment *j*; β_i_ is the effect of block *i*; T_j_ is the effect of dietary treatment *j*, and e_ijk_ is the random residual error in treatment *j* for block *i*.

Residual distributions from general linear models were studied. Assumptions for normality, linearity, and heteroscedasticity were tested. Box–Cox transformation was used to estimate transformation parameters [21]. A ”power transformation” on Y was used to satisfy the normality assumption. Estimates are expressed as the mean ± Standard Error. When significance was found, *post hoc* Tukey multiple comparisons were performed using the “emmeans” package [22]. A graph was plotted using “ggplot 2” package [23]. All statistical analyses were performed using the R program version 4.3 [24]. A significance level was defined at p < 0.05.

## Results

### *In vitro* protein digestibility

The percentages of *in vitro* dry matter and protein digestibility of soybean meal protein did not differ significantly (p > 0.05) between procreation and protease. However, the results illustrated that pancreatin tended to have higher (p = 0.07) enzyme activity than protease (Table-2).

**Table-2:** Enzyme activity and *in vitro* digestibility of dry matter and soybean meal protein in the presence of pancreatin or protease enzyme.

Item, %	Pancreatin^1/^	Protease^2/^	p-value
Enzyme activity (U/g)	13,569.20 ± 106.66	12,808.52 ± 274.26	0.071
*In vitro* dry matter digestibility	81.77 ± 2.82	82.77 ± 1.02	0.592
*In vitro* protein digestibility	90.42 ± 0.16	90.40 ± 0.23	0.933

1/ Pancreatin: protease, a standard known enzyme activity.

2/ Protease: trial protease used in the experiment

### Growth performance

**Figure-1:**
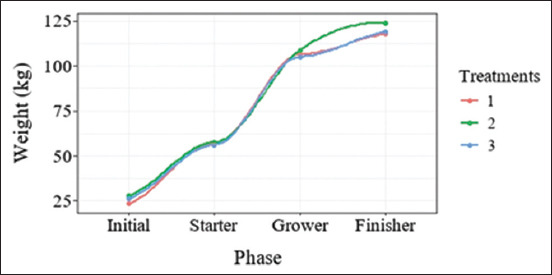
Growth curves of starter-to-finisher pigs fed three different treatments. The body weight of T2 pigs was significantly higher (p < 0.05) than those of T1 and T3 pigs at the initial, starter, grower, and finisher phases. The orange, green, and blue lines represent the body weights of the T1, T2, and T3 pigs, respectively.

The graph in Figure-1 shows a progressive increase in phases according to BW. The results showed that the BW of T2 pigs was higher (p < 0.05) than that of T1 and T3 pigs at the starter and finisher phases but lower (p < 0.05) than that of T1 and T3 pigs at the grower phase. The ADG of T3 pigs at the start, grower, finisher, and overall stage was lower (p < 0.05) than that of T1 and T2 pigs. ADFI of T3 pigs at the start, grower, and overall stage was lower (p < 0.05) than that of T1 and T2 pigs. However, the percentage of T3 pigs was lower than that of T1 pigs only during the finisher phase. The F:G of T2 pigs at starter was higher (p < 0.05) than that of T1 and T3 pigs, while it was lower (p < 0.05) than that of T1 at the grower phase. F:G of T3 pigs at the finisher phase was higher (p < 0.05) than that of T1 and T2 pigs. G:F of T2 pigs was at starter phase lower (p < 0.05) than that of T1 and T3 pigs but higher at the grower phase (p < 0.05) than that of T1. The F:G of T1 and T2 pigs were at finisher phase higher (p < 0.05) than that of T3 pigs (Table-3).

**Table-3:** Performance of pigs fed diets with protease supplementation.

Item	Treatment			p-value

	T1^1/^	T2	T3	
Starter phase				
Initial body weight (kg)	23.36 ± 0.62^c^	27.90 ± 0.57^a^	20.45 ± 0.38^b^	<0.010
Adjusted final body weight, (kg)^2/^	57.90 ± 0.62^b^	62.01 ± 0.61^a^	59.20 ± 0.61^b^	<0.010
DIH, days^3/^	43.02 ± 0.08^a^	42.71 ± 0.09^b^	40.83 ± 0.07^c^	0.001
ADG, kg/day^4/^	0.84 ± 0.02^a^	0.85 ± 0.02^a^	0.77 ± 0.01^b^	<0.010
ADFI, kg/day^5/^	1.61 ± 0.01^b^	1.65 ± 0.01^a^	1.53 ± 0.01^c^	<0.010
F:G^6/^	2.12 ± 0.01^b^	2.16 ± 0.01^a^	2.12 ± 0.01^b^	0.021
G:F^7/^	0.47 ± 0.01^a^	0.46 ± 0.01^b^	0.47 ± 0.01^a^	<0.010
Grower phase				
Initial body weight (kg)	57.90 ± 0.62^b^	62.01 ± 0.61^a^	59.20 ± 0.61^b^	<0.010
Adjusted final body weight (kg)	107.77 ± 0.90^a^	103.75 ± 1.21^b^	106.26 ± 0.87^ab^	0.029
DIH, days	61.07 ± 1.31^b^	72.50 ± 1.44^a^	64.26 ± 1.27^b^	<0.001
ADG, kg/day	0.81 ± 0.01^a^	0.77 ± 0.01^ab^	0.77 ± 0.01^b^	0.022
ADFI, kg/day	2.34 ± 0.01^a^	2.30 ± 0.02^a^	2.27 ± 0.04^b^	<0.010
F:G	3.06 ± 0.02^a^	2.97 ± 0.02^b^	3.03 ± 0.02^ab^	0.011
G:F	0.33 ± 0.01^ab^	0.34 ± 0.01^a^	0.33 ± 0.01^b^	0.045
Finisher phase				
Initial body weight (kg)	107.77 ± 0.90^a^	103.75 ± 1.21^b^	106.26 ± 0.87^ab^	0.029
Adjusted final body weight (kg)	120.58 ± 1.15	123.15 ± 1.50	119.15 ± 1.10	0.110
DIH, days	22.96 ± 2.36^b^	44.01 ± 2.36^a^	26.26 ± 2.28	<0.001
ADG, kg/day	0.90 ± 0.06^ab^	1.04 ± 0.09^a^	0.77 ± 0.06^b^	0.004
ADFI, kg/day	2.65 ± 0.01^a^	2.61 ± 0.01^b^	2.61 ± 0.01^b^	0.001
F:G	3.24 ± 0.07^b^	3.02 ± 0.10^b^	4.13 ± 0.07^a^	<0.001
G:F	0.34 ± 0.01^a^	0.33 ± 0.01^a^	0.26 ± 0.01^b^	<0.010
Overall				
Initial body weight (kg)	23.36 ± 0.62^c^	27.90 ± 0.57^a^	20.45 ± 0.38^b^	<0.010
Adjusted final body weight (kg)	119.27 ± 1.32	123.59 ± 1.31	119.06 ± 1.29	0.219
DIH, days	97.08 ± 1.82	100.29 ± 1.84	98.80 ± 1.77	0.460
ADG, kg/day	0.82 ± 0.01^a^	0.79 ± 0.01^ab^	0.76 ± 0.01^b^	0.013
ADFI, kg/day	2.03 ± 0.01^a^	2.05 ± 0.01^a^	1.98 ± 0.01^b^	<0.001
F:G	2.68 ± 0.02^b^	2.69 ± 0.02^a^	2.69 ± 0.02^a^	0.035
G:F	0.38 ± 0.01	0.38 ± 0.01	0.38 ± 0.01	0.124

^a,b,c^Within a row, means ± se without a common superscript differ significantly (p < 0.05).

1/ T1: basal diet; T2: The basal diet supplemented with 240 ppm protease reduced by 50 kcal/kg ME plus 1% CP; T3: The basal diet supplemented with 240 ppm protease reduced by 100 kcal/kg ME plus 2% CP.

2/ Adjusted final body weight: Final body weight covariated using the initial body weight.

3/ DIH: Day in Herd.

4/ ADG: Average Daily Gain.

5/ ADFI: Average Daily Feed Intake.

6/ F: G: Feed-to-Gain Ratio as feed efficiency.

7/ G: F: Gain-to-Feed Ratio as gain efficiency

## Discussion

This study investigated the effects of proteases included in diets in which the consideration of nutrient moderation was considered. Treatment groups were determined by reducing ME and CP intake from the basal diet when protease was added. The effects of exogenous proteases were examined by *in vitro* digestibility of dry matter and soybean meal protein, and the *in vivo* productivity of starter-to-finisher pigs. This study found that supplemental proteases were potentially digestible equivalent to endogenous enzymes, thereby maintaining growth performance, feed (F:G), and gain (G:F) efficiency that were compensable for 50 kcal/kg ME and 1% CP reduction, respectively.

The results of an *in vitro* study on dry matter and protein digestibility revealed insignificant differences in the levels and activities of supplemental protease and pancreatin, which represent endogenous protease. To the best of our knowledge, nutrient digestibility in pigs can be improved by supplementing exogenous proteases, most likely through hydrolyzing peptide bonds before or after the incorporation of specific amino acids into peptides and amino acids [1]. Moreover, exogenous proteases may enhance nutrient digestion by offering broader substrate specificity and reducing antinutritional effects [25], assessing endogenous proteases [1], improving the integrity of amino acid transporters and tight junctions [26], and minimizing the loss of endogenous amino acids through digestive enzyme secretion or mucin synthesis [27]. These factors contribute to enhanced apparent ileal digestibility, amino acid retention, and absorption [1, 28].

This study found insignificant differences in growth performance between T1 and T2 pigs throughout the experiment, whereas T3 pigs exhibited slower growth, which implied that reductions of 50 kcal/kg ME and 1% CP did not affect feed and gain efficiency. These results are consistent with the findings of Nguyen *et al*. [29], who reported that weight gain and ADG were not affected by supplementation in grower pigs fed with protease in low- or high-protein diets. However, these results are contrary to a previous report by Wang *et al*. [30] in which ADG was increased after feeding nursery pigs a keratinase-supplemented diet. This may be due to differences in enzyme-specific feed ingredients, growth phase, and gut development, allowing starter-to-finisher pigs to use dietary nutrients more efficiently than nursery pigs. Nevertheless, the growth rate of T3 pigs was low. This indicates that 100 kcal/kg ME and 2% CP reduced from the basal diet may lead to insufficient nutrient levels.

Although this study observed fluctuations in ADFI following the growth phases and DIH, an insignificant difference was observed overall among the treatments. According to a previous study by Lee *et al*. [8], ADFI was insignificantly different after the grower pigs were fed a protease-supplemented diet. This was most likely explained by the natural variance of the initial and final BWs of pigs, which are the primary determinants of DIH and ADFI. Accordingly, the gain efficiency of T2 pigs was improved at the grower and finisher phases while that of T3 pigs at the finisher phase was significantly lower; however, overall, a significant difference was not found in T3 compared with T1 and T2 pigs. Previous studies by Lee *et al*. [8] and Kim *et al*. [12] have shown that a low-CP diet supplemented with protease could increase the gain efficiency, obvious total tract digestibility of protein, and energy of finisher pigs. Our study demonstrated that a T2 diet conserved growth and feed efficiency during the grower, finisher, and overall. However, the T3 diet showed improvement in growth and feed efficiency during the starter and grower phases but deterioration during the finisher phase. Nevertheless, 100 kcal/kg ME and 2% CP reduction from the basal diet may lead to insufficient nutrients for growth, which is consistent with a previous report by Nguyen *et al*. [29] showing that 1.21% CP and 98 kcal/kg (0.41 MJ/kg) reduced from the control diet contributed to lower growth performance and feed efficiency.

Although numerous studies have suggested the benefits of reducing dietary ME in broilers [14, 15, 31, 32], relatively few studies have been conducted on pigs. In addition to augmenting protein digestibility [1, 13], exogenous proteases can enhance dietary ME by as much as 7%. By breaking down proteins into a chyme complex, the enzyme increases the availability of endogenous enzymes to the surface area of fats or dietary energy sources [14, 15]. In broilers, protease supplementation increased the apparent ME and net energy by 73 and 107 kcal/kg, respectively [32]. Moreover, improved protein digestibility not only enhances amino acid retention and energy utilization through “protein-sparing effect” [33] but also reduces nitrogen in the hindgut [13] and promotes gut health [34] in weaned pigs fed protease. In addition, protease supplementation improves the uniformity of pig weights, with lower variability in BW in supplemented pigs compared with control pigs. This result was similar to that of the broiler trial, in which enhanced amino acid digestibility reduced variability in digestive capacity and consequently increased flock uniformity [31]. These findings suggest that a reduction of both 1% CP and 50 kcal/kg ME from the basal diet in the present study is equivalent to a 100 kcal ME reduction, as suggested by Cowieson *et al*. [32], which compensates for the maintenance of gain efficiency.

## Conclusion

Protease supplementation is effective for nutrient digestibility through *in vitro* digestion of soybean meal. The *in vivo* study found that protease supplementation at 240 ppm was compatibly compensatory to a diet containing 50 kcal/kg ME and 1% CP reduction, allowing starter-to-finisher pigs to maintain better growth performance. A limitation of this study is that the experiment was performed in a large commercial setting and may not be well-controlled comparable to a laboratory setting. Future studies should investigate the dose-response relationship of protease inclusion levels in starter-to-finisher diets. In addition, the levels of protease supplemented in diets may be studied in nursery pigs, replacement gilts, gestating, and lactating sows.

## Authors’ Contributions

NH: Experimental design and conceptual framework. PS, SJ, AK, SC, CK, and NH: Performed the experiment and drafted the manuscript. PS, AK, and NH: Data analysis. All authors have read, reviewed, and approved the final manuscript.
